# Structure Prediction and Genome Mining‐Aided Discovery of the Bacterial C‐Terminal Tryptophan Prenyltransferase PalQ

**DOI:** 10.1002/advs.202307372

**Published:** 2023-12-07

**Authors:** Azusa Miyata, Sohei Ito, Daisuke Fujinami

**Affiliations:** ^1^ Graduate Division of Nutritional and Environmental Sciences University of Shizuoka 52‐1 Yada, Suruga‐ku Shizuoka 422‐8526 Japan

**Keywords:** genome mining, post‐translational modification, prenylation, structure prediction

## Abstract

Post‐translational prenylations, found in eukaryotic primary metabolites and bacterial secondary metabolites, play crucial roles in biomolecular interactions. Employing genome mining methods combined with AlphaFold2‐based predictions of protein interactions, PalQ , a prenyltransferase responsible for the tryptophan prenylation of RiPPs produced by *Paenibacillus alvei, is identified*. PalQ differs from cyanobactin prenyltransferases because of its evolutionary relationship to isoprene synthases, which enables PalQ to transfer extended prenyl chains to the indole C3 position. This prenylation introduces structural diversity to the tryptophan side chain and also leads to conformational dynamics in the peptide backbone, attributed to the *cis/trans* isomerization that arises from the formation of a pyrrolidine ring. Additionally, PalQ exhibited pronounced positional selectivity for the C‐terminal tryptophan. Such enzymatic characteristics offer a toolkit for peptide therapeutic lipidation.

## Graphical abstract

Tryptophan prenyltransferase PalQ is a novel member of the isoprene synthase domain superfamily. The C3 prenylation of the tryptophan side chain, mediated by PalQ, alters the conformational dynamics of the peptide backbone.

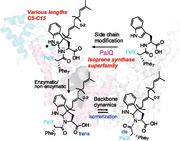

## 1 Introduction

Prenylation is the covalent attachment of an isoprenyl group to a protein, and is one of the most common post‐translational modifications.^[^ ^1^ ^]^ In mammals, up to 2% of cellular proteins undergo prenylation.^[^ ^2^ ^]^ These modifications mediate both protein‐membrane and protein–protein interactions.^[^ ^1^ ^]^ In eukaryotes, prenylation is catalyzed by four distinct enzymes.^[^ ^3^ ^]^ Farnesyl transferase (FTase) and Geranylgeranyl transferases 1 (GGTase1) and 3 (GGTase3) all recognize the C‐terminal CaaX motif of acceptor proteins, where “C” stands for cysteine, “a” for an aliphatic residue, and “X” for any amino acid at the C‐terminus.^[^ ^4^ ^]^ The nature of the X amino acid residue determines that prenyltransferase will act.^[^ ^5^ ^]^ In contrast, Geranylgeranyl transferase 2 (GGTase2) recognizes less defined C‐termini, such as the CC and CXC sequences. FTase and GGTases transfer C15 and C20 isoprenoid‐moieties from farnesyl diphosphate (FPP) and geranylgeranyl diphosphate (GGPP) to the cysteine(s), forming a covalent bond between the cysteine thiol and the C1 carbon of the isoprenoid moiety (Figure S1, Supporting Information). After prenylation, the C‐terminal aaX is often cleaved by proteases, and then the carboxyl group of the isoprenylcysteine is methylated by a carboxyl‐methyltransferase.^[^ ^6^ ^]^ All four prenyltransferases are heterodimers, each comprising an α and a β subunit.^[^ ^7^ ^]^

Notably, post‐translational prenylation is not exclusive to eukaryotes; it is also evident in bacteria, particularly within cyanobacteria.^[^ ^8^, ^9^ ^]^ In bacteria, prenylation is observed on secondary metabolites like ribosomally synthesized and post‐translationally modified peptides (RiPPs), and contributes to increase the structural diversity of this natural product family.^[^ ^10^ ^]^ Such bacterial prenylations are usually catalyzed by cyanobactin prenyltransferases (PTases). In contrast to eukaryotic prenyltransferases, PTases function as monomers that adopt an α+β barrel fold, and belong to the F‐family of prenyltransferases (InterPro: IPR031037). The barrel cavity of PTases is tailored to accommodate donor isoprenyl substrates, with a preference for shorter isoprenyl diphosphates, such as dimethylallyl (DMAPP, C5) and geranyl (GPP, C10) diphosphates. Both forward and reverse prenylations have been identified (Figure S1, Supporting Information). Unlike the donor substrate recognition site, the acceptor peptide binding site is solvent‐exposed, and lacks a well‐defined sequence motif for acceptor substrate recognition. To date, various PTases have been identified, mediating prenylations on amino acids such as Ser/Thr,^[^ ^11^, ^12^ ^]^ Tyr,^[^ ^13^, ^14^, ^15^, ^16^ ^]^ Trp,^[^ ^16^, ^17^ ^]^ His,^[^ ^18^ ^]^ and Arg,^[^ ^19^ ^]^ as well as the backbones at the C‐^[^ ^20^ ^]^ and N‐termini.^[^ ^21^, ^22^ ^]^ Typically, these modifications occur in cyclic peptides. The broad specificity of PTases highlights their potential as tools for peptide therapeutic lipidation, which could potentially amplify their therapeutic properties.

Beyond cyanobacteria, prenylations of tryptophan have also been identified in *Bacillus* bacteria.^[^ ^23^ ^]^ ComX is a RiPP involved in *Bacillus* bacterial quorum sensing and is matured through geranylation or farnesylation.^[^ ^24^ ^]^ ComQ catalyzes forward prenylation at the indolic C3 position of an internal tryptophan within the ComX peptide sequence. Following this prenylation, the backbone amide nitrogen of the prenylated tryptophan cyclizes onto the indole ring at C2, generating a tricyclic pyrroloindole structure (Figure S1, Supporting Information). This prenylated precursor peptide undergoes further cleavage by unknown proteases to yield the mature ComX. Interestingly, ComQ is part of the isoprene synthase domain superfamily, suggesting a distinct evolutionary lineage compared to cyanobactin PTases.^[^ ^25^ ^]^ While ComQ possesses the first aspartate‐rich motif (FARM), it noticeably lacks the second aspartate‐rich motif (SARM), even though both motifs are characteristic of isoprene synthases (Figure S2, Supporting Information). Previous mutational studies suggested that the FARM and pseudo‐SARM of ComQ play crucial roles in binding the donor prenyl diphosphate and the acceptor ComX substrates, respectively.^[^ ^25^, ^26^ ^]^

In this study, we report a tryptophan prenyltransferase found in the *Paenibacillus alvei* RiPP biosynthetic gene cluster. This prenyltransferase and RiPP were named PalQ and PalX, respectively. Although PalQ had previously been annotated as a terpene synthase due to its low sequence similarity with known prenyltransferases, the AlphaFold2‐predicted interaction between PalQ and PalX led us to hypothesize that PalQ functions as a peptide prenyltransferase. Our in vitro analysis confirmed that PalQ prenylates the indolic C3 in a forward direction, and further highlighted its stringent selectivity for the tryptophan residue at the C‐terminus. NMR analysis of the prenylated peptide revealed a tricyclic pyrroloindole structure with a mix of *trans‐* and *cis‐* peptide backbone configurations. Molecular dynamics simulations of the PalQ‐PalX complex model suggested that the FARM motif coordinates a singular divalent ion, favoring forward prenylation over reverse. We believe that the C‐terminal prenylation mediated by PalQ could be applied to late‐stage peptide therapeutic lipidations, while causing minimal structural alterations of the primary structure.

## 2 Results and Discussion

### 2.1 Structure‐Aided Genome Mining

**Figure 1:**
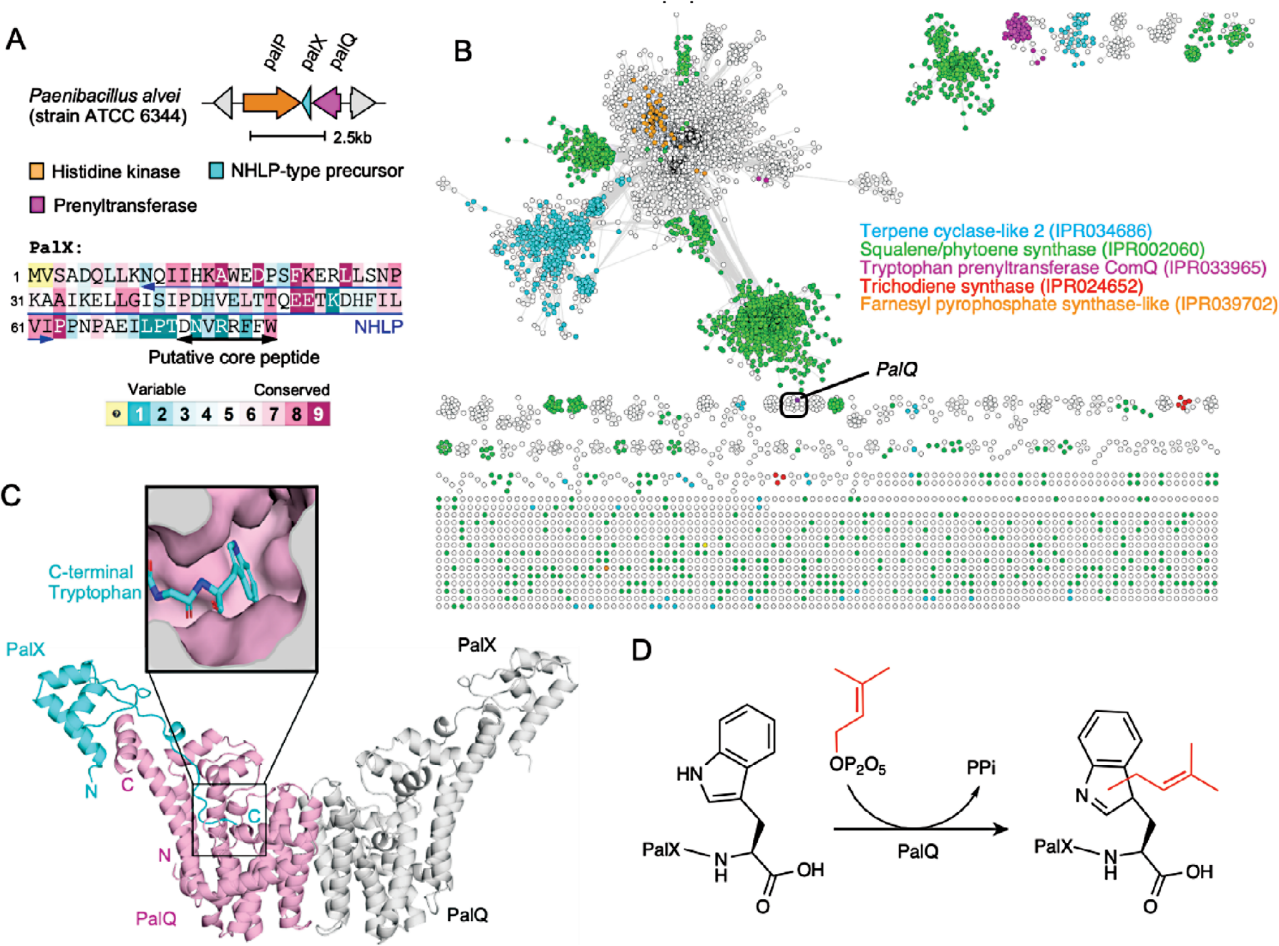
Structure‐aided genome mining. A) Graphical representation of the *P. alvei* PalX biosynthetic gene cluster. The amino acid sequence of PalX is shown, with the NHLP and core regions underlined. The bar denotes the amino acid conservation score derived from the Consurf analysis. B) Sequence similarity network for the isoprene synthase domain superfamily. The IPR008949 domain was used to query the EFI‐EST tools, and the results were visualized in Cytoscape.^[^ ^32^ ^]^ Nodes were clustered based on an alignment score threshold of 30. The cluster that includes PalQ is highlighted with a black box. C) AlphaFold2 model of the PalQ‐PalX complex. The inset provides a closer view of the putative active site of PalQ. D) Proposed reaction scheme catalyzed by PalQ.

The genus *Paenibacillus* comprises Gram‐positive bacteria that secrete a variety of bioactive secondary metabolites, including RiPPs, which have promising applications in medical, food, agricultural, and industrial fields.^[^ ^27^ ^]^ *Paenibacillus alvei*, a facultative, endospore‐forming mesophilic bacterium, is recognized for its role as a secondary infector in European Foulbrood disease.^[^ ^28^ ^]^ We identified a RiPP biosynthetic gene cluster within the *Paenibacillus alvei* genome (Genome ID: AMBZ01000001), using antiSMASH.^[^ ^29^ ^]^ The cluster comprises three genes: a histidine kinase (PalP), a nitrile hydratase leader peptide (NHLP)‐type precursor peptide (PalX), and a prenyltransferase (PalQ) (**Figure** **1**A). This gene cluster is conserved across various *Paenibacillus* bacteria (Figure S3, Supporting Information). Upon analyzing the amino acid sequence of PalX with Consurf,^[^ ^30^ ^]^ we found that both the NHLP domain and the C‐terminal tryptophan residue are conserved. The NHLP domain typically binds to post‐translational modifying enzymes, and is subsequently cleaved by proteases to produce mature RiPPs.^[^ ^31^ ^]^ To investigate the digestion of PalX, we treated the recombinant PalX with a *P. alvei* cell lysate. The MALDI‐TOF‐MS analysis revealed that the digestion occurred between T72 and D73 (Figure S4, Supporting Information), leading us to propose DNVRRFFW as the core peptide.

Previously, PalQ was identified as a terpene synthase (Uniprot ID: K4ZRG6) that belongs to the isoprene synthase domain superfamily (InterPro: IPR008949). This superfamily includes several prominent subfamilies, such as Terpene cyclase‐like2 (InterPro: IPR034686), Squalene/phytoene synthase (IPR002060), Tryptophan prenyltransferase ComQ (IPR033965), Trichodiene synthase (IPR024652), and Farnesyl pyrophosphate synthase‐like (IPR039702). These subfamilies are each grouped in the Sequence Similarity Network for the isoprene synthase domain superfamily, as constructed using EFI‐EST^[^ ^33^ ^]^ (Figure 1B). Notably, within this network, PalQ forms a distinct cluster, highlighting its remote relationship to these subfamilies (Table S1 and, Figures S5 and S6, Supporting Information). To obtain structural and functional insights, we employed AlphaFold2 prediction.^[^ ^34^, ^35^ ^]^ PalQ was predicted to form a homodimer, as well as a complex with PalX (Figure 1C; Figure S7, Supporting Information). The formation of the homodimer and the complex were experimentally confirmed using size exclusion chromatography and native agarose gel electrophoresis, respectively (Figure S8, Supporting Information). Examining the predicted structure, we noticed that the C‐terminal tryptophan of PalX inserts into the putative active site of PalQ (Figure 1C, *inset*). This observation led us to hypothesize that PalQ acts as a prenyltransferase, prenylating the C‐terminal tryptophan of PalX (Figure 1D).

### 2.2 PalQ Transfers Various Lengths of Isoprenyl Chains to PalX

**Figure 2:**
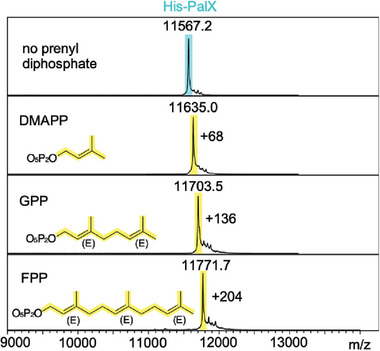
Donor substrate analysis. A) MALDI‐TOF‐MS spectra of ComX after incubations with different donor prenyl diphosphates. The theoretical average m/z for the N‐terminally Met‐cleaved His‐PalX is 11 566, highlighted in cyan.

To test our hypothesis, we incubated PalQ with PalX in the presence of donor prenyl diphosphates with different isoprenyl lengths (C5‐DMAPP, C10‐GPP, and C15‐FPP). The MALDI‐TOF‐MS detected mass shifts of +68, +136, and +204, which correspond to mono‐dimethylallylation, geranylation, and farnesylation, respectively (**Figure** **2**). We then assessed the kinetics of PalQ for DMAPP, GPP, and FPP. Due to the poor water solubility of GPP and FPP, we were unable to produce the asymptotic flattening of their Michaelis–Menten curves (Figure S9, Supporting Information). Nonetheless, our data indicated a trend where diphosphates with longer isoprenyl chains serve as more potent substrates. For the sake of experimental simplicity, we chose DMAPP for the subsequent in vitro assays.

### 2.3 NHLP Domain of PalX Enhances Prenylation by PalQ

PalX possesses an N‐terminal NHLP domain. To investigate the role of the NHLP domain in prenylation, we synthesized the core peptide, DNVRRFFW, and compared its activity to that of the full‐length PalX. The enzymatic assay was conducted by incubating PalQ with DMAPP and MgCl_2_, using various concentrations of either PalX or the core peptide. We found that under conditions with limited substrate, full‐length PalX was a more effective substrate than the core peptide (Figure S10, Supporting Information), suggesting that the NHLP domain enhances the interaction with PalQ. However, it is crucial to note that the core peptide still showed activity, indicating that the NHLP domain is not absolutely essential. We then used the octapeptide for the subsequent NMR analysis of the prenylated product.

### 2.4 NMR Analysis Reveals a Tricyclic Pyrroloindole Structure

**Figure 3:**
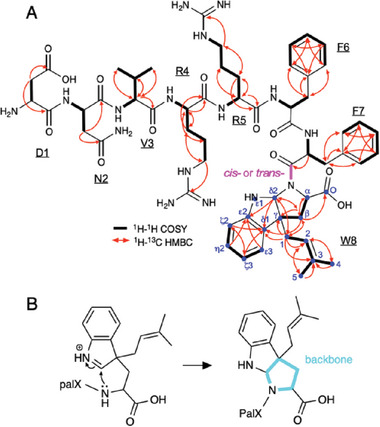
Chemical structure and NMR characterization of the prenylated octapeptide DNVRRFFW. A) ^1^H─^1^H COSY and ^1^H─^13^C HMBC correlations are illustrated on the chemical structure. The peptide bond between F7 and W8 is depicted in magenta, while carbon atoms of the prenylated W8 are indicated in blue. B) Schematic representation of the tricyclic pyrroloindole formation. The peptide backbone pyrrolidine ring is emphasized with a thick cyan line.

The chemical structure of the prenylated octapeptide (^1^DNVRRFFW^8^) was determined by NMR, using ^1^H─^1^H DQF‐COSY, ^1^H─^1^H TOCSY, ^1^H─^1^H ROESY, ^1^H─^13^C gHSQC, and ^1^H─^13^C HMBC (Figures S11–S16, Supporting Information). We identified six amino acid spin systems, which were assigned as D1, N2, V3, R4, R5, and F6. The ^1^H and ^13^C chemical shifts of R4 and R5 showed significant signal overlapping, implying a similar magnetic environment. Additionally, we detected two distinct sets of spin systems, which we assigned as F7/F7’ and W8/W8’. The up‐field shifts observed in β and δ2 (corresponding to the indole C2) protons of W8 (δ_Hβ_ = 2.14 and 2.48 ppm; δ_Hδ2_ = 5.27 ppm) and W8’ (δ_Hβ_ = 2.12 and 2.54 ppm; δ_Hδ2_ = 5.27 ppm), in comparison with the standard Trp (average δ_Hβ_ = 3.12 and 3.17; δ_Hδ2_ = 7.13 ppm), indicate the loss of aromaticity in the pyrrole ring. Furthermore, W8 and W8’ were missing the backbone NH signals. These observations indicated the formation of a tricyclic pyrroloindole structure.^[^ ^23^ ^]^ The pyrrolidine ring in the peptide backbone could cause *cis/trans* isomerization of the peptide bond, analogous to the proline pyrrolidine ring. Based on the ^13^C chemical shifts,^[^ ^36^ ^]^ W8 (δ_Cβ_ = 42.3 and δ_Cγ_ = 57.7 ppm) appears to be in a *trans‐*configuration, while W8’ (δ_Cβ_ = 41.8 and δ_Cγ_ = 60.4 ppm) is in a *cis‐*configuration in the F7‐W8 peptide bond (**Figure** **3**). The differences in the chemical shifts between the two configurations might be attributable to the ring current effects of the aromatic rings in F7 and W8. The NMR peak intensities suggested that the trans‐configuration is the predominant form.

We observed two sets of NMR signals corresponding to the prenyl group oriented in the forward direction, as represented by W8 (δ_C_ = 37.1, 120.9, 138.7, 19.3, 27.2 ppm; δ_H_ = 2.30, 5.04, 1.37, 1.58 ppm) and W8’ (δ_C_ = 37.0, 121.1, 138.7, 19.4, 27.2 ppm; δ_H_ = 2.41, 5.00, 1.44, 1.58 ppm). The ^1^H─^13^C HMBC correlations, H1─C_β_, C1─H_β_, H1─C_γ_, H1─C_δ2_, and H1─C_δ1_, indicated a C─C bond between the C1 and C_γ_ (corresponding to the indole C3) carbons (Figure 3). The absence of a ^1^H signal at the γ position further supported this linkage.

### 2.5 Structural Insights into Forward Prenylation

**Figure 4:**
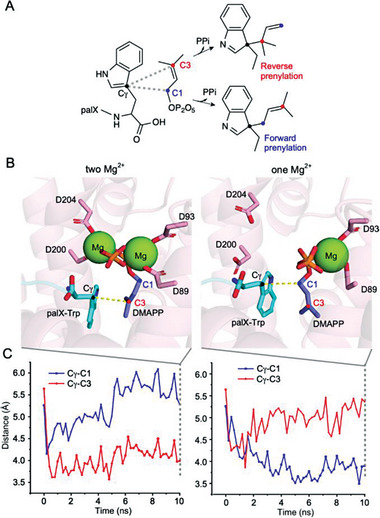
Molecular dynamic simulations of the PalQ‐PalX‐DMAPP complexes with one or two magnesium ions. A) Reaction schemes illustrating reverse and forward prenylations. The blue and red circles represent the C1 and C3 carbons, respectively. B) Close‐up views of the active sites after a 10 ns simulation, comparing the complex model with two magnesium ions (left) to the one with a single magnesium ion (right). C) Distances between Cγ─C1 (blue line) and Cγ─C3 (red line) over the simulation time. Plots correspond to the simulations with two Mg ions (left) and one Mg ion (right).

Polypeptide prenylation is thought to proceed through a dissociative electrophilic substitution mechanism,^[^ ^8^, ^13^, ^14^, ^37^ ^]^ in which the removal of the pyrophosphate group from a prenyl diphosphate generates an allylic carbocation at either the C1 or C3 positions. An attack on the C1 allylic carbocation by a nucleophile in a prenyl acceptor results in forward prenylation. Conversely, targeting the C3 allylic carbocation results in reverse prenylation (**Figure** **4**A).

Members of the isoprene synthase domain superfamily typically coordinate divalent ion(s) at the FARM and SARM.^[^ ^38^ ^]^ These metals also coordinate the substrate isoprenyl diphosphate, providing an ionizing forcethat facilitates the dissociation of the pyrophosphate moiety from a prenyl diphosphate. To investigate the influence of metals on the PalQ reaction, we conducted prenylation assays with various divalent metals, including MgCl_2_, CaCl_2_, FeSO_4_, MnCl_2_, and ZnCl_2_, and found that PalQ functions as a prenyltransferase dependent on either MgCl_2_ or MnCl_2_ (Figure S17, Supporting Information).

To gain structural insight into the forward prenylation catalyzed by PalQ, we performed molecular dynamics simulations using GENESIS.^[^ ^39^ ^]^ Our initial computational model was constructed by manually integrating DMAPP into the AlphaFold2‐predicted PalQ‐PalX model, referring to the crystal structure of an isoprene synthase (PDBID 3OYR).^[^ ^40^ ^]^ Magnesium ions were introduced using the ProBiS server,^[^ ^41^ ^]^ resulting in two distinct metal‐binding patterns. One involves the coordination of two magnesium ions at both FARM (D89 and D93) and SARM (D200 and D204), while the other coordinates a single magnesium ion at SARM (D200 and D204) (Figure 4B). The one‐Mg and two‐Mg complexes were both subjected to energy minimization to remove potential artifacts, and then analyzed through short‐duration molecular dynamics simulations for 10 ns. The MD trajectory analyses revealed distinct orientations of the dimethylallyl chain. In the single magnesium ion model, the closest atomic pair comprised the Cγ and C1 carbons. In contrast, in the two‐Mg model, the C3 carbon was nearest to the Cγ carbon (Figure 4C). Based on these findings, we propose that the one‐Mg model accounts for the forward prenylation, as characterized by NMR.

Both D200 and D204 at the SARM are conserved among PalQ homologues (Figure S6, Supporting Information). However, the double mutant PalQ D200A/D204A retained activity in the endpoint prenylation assay, suggesting these residues are not essential for prenylation activity (Figure S18, Supporting Information).

### 2.6 PalQ Displays Specificity for Tryptophan at the C‐Terminus

**Figure 5:**
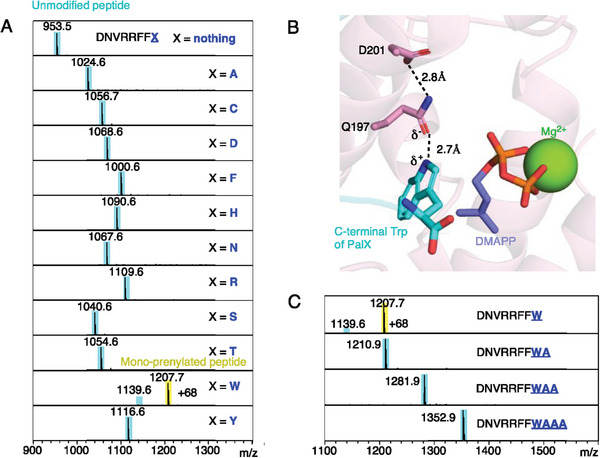
Analysis of acceptor substrates. A) MALDI‐TOF‐MS spectra of various core peptides after incubation with DMAPP. Data were acquired at the endpoints of the prenylation assay. Peaks corresponding to unmodified and mono‐prenylated peptides are highlighted in cyan and yellow, respectively. B) Close‐up view of the active site in PalQ. Hydrogen bonds are indicated by dashed lines. C) MALDI‐TOF‐MS spectra of DNVRRFFW variants with extended alanine sequences at the C‐terminus.

According to the electrophilic substitution mechanism, amino acid prenylations predominantly occur on electron‐rich atoms (Figure S1, Supporting Information). To explore prenylation beyond tryptophan, we designed a series of core peptides, DNVRRFFX, where “X” represents various electron‐rich amino acids, and subjected them to prenylation assays. Only the peptide DNVRRFFW exhibited mono‐prenylation, indicating that PalQ acts specifically as a tryptophan‐targeting prenyltransferase (**Figure** **5**A). In the one‐magnesium PalQ‐PalX model, the highly conserved Q197, adjacent to the SARM motif, recognizes the indole NH through a hydrogen bond, potentially contributing to the specificity for tryptophan (Figure 5B). Moreover, this interaction could increase the nucleophilicity of the Cγ carbon, by either deprotonating it or introducing a partial positive charge to the indole nitrogen.^[^ ^42^ ^]^

To assess the positional preference of tryptophan, we synthesized DNVRRFFW variants that extended the C‐terminus by different lengths of alanine. The results showed that adding extra alanine residues at the C‐terminus abolished the prenylation activity (Figure 5C). This highlights the importance of precisely positioning the tryptophan at the C‐terminus for it to serve as a substrate for prenylation.

### 2.7 Structural Comparison Between PalQ and ComQ

We have confirmed that PalQ can transfer a C15 farnesyl chain, a feature also reported for *Bacillus subtilis* ComQ but not observed in cyanobactin PTases (Figure S1, Supporting Information).^[^ ^43^, ^44^ ^]^ Molecular dynamics simulations were performed to visualize the binding within the modeled PalQ‐PalX and ComQ‐ComX complexes in the presence of FPP (Figures S19 and S20, Supporting Information). The donor substrate binding site of PalQ offers ample space to accommodate the C15 FPP (Figures S20A,B, Supporting Information), suggesting that this expansive cavity is the structural basis for PalQ's tolerance of donor substrates with elongated prenyl chains. We further tested whether C20 GGPP could serve as a donor substrate; however, it was not accepted (Figure S21, Supporting Information). Notably, the binding site of *B. subtilis* ComQ differs from that of PalQ in the shape and orientation of its cavity, which is oriented toward the ComQ dimer interface.

PalQ specifically prenylates the C‐terminal tryptophan, while *Bacillus* ComQ prenylates internal tryptophan residues.^[^ ^43^ ^]^ To gain structural insights into these positional preferences, we compared the acceptor substrate binding surfaces of PalQ and ComQ (Figure S20C,D, Supporting Information). During molecular dynamics simulations, the carboxyl group at the C‐terminus of PalX occasionally forms an electrostatic interaction with K39 of PalQ. Nevertheless, amino acid sequence analysis shows that K39 is not highly conserved among PalQ homologs (Figure S6, Supporting Information). Therefore, we propose that the differences in positional preferences are more attributable to the shape of the acceptor substrate binding surfaces than to specific non‐covalent interactions between the acceptor peptides and the enzymes.

## 3 Conclusion

In conclusion, through traditional genome mining approaches supplemented by AlphaFold2‐informed protein interaction models, we discovered a novel protein prenyltransferase, PalQ. Although PalQ shares a low pairwise sequence identity (< = 25%) with the previously characterized tryptophan prenyltransferase ComQ, it still facilitates similar tryptophan Cγ prenylation in the forward direction.^[^ ^24^ ^]^ After prenylation, the product undergoes either enzymatic or nonenzymatic tricyclic pyrroloindole formation. NMR structural analysis of the peptide revealed the cis/trans isomerization of the peptide backbone. An in silico analysis suggested that magnesium coordination at the FARM is responsible for selective forward prenylation over reverse prenylation. PalQ stands out among bacterial prenyltransferases because it exclusively prenylates the C‐terminal residue, similar to eukaryotic prenyltransferases. Additionally, PalQ is capable of catalyzing the transfer of a farnesyl group, paralleling the function of its eukaryotic counterparts. Therefore, PalQ might be instrumental in facilitating chemo‐enzymatic peptide lipidation that mimics human‐type prenylation.

## Conflict of Interest

The authors declare no conflict of interest.

## Supporting information

Supporting Information

## Data Availability

The data that support the findings of this study are available in the supplementary material of this article.

## References

[1] H. Jiang , X. Zhang , X. Chen , P. Aramsangtienchai , Z. Tong , H. Lin , Chem. Rev. 2018, 118, 919. 29292991 10.1021/acs.chemrev.6b00750 PMC5985209

[2] W. W. Epstein , D. Lever , L. M. Leining , E. Bruenger , H. C. Rilling , Proc. Natl. Acad. Sci. USA 1991, 88, 9668. 1946384 10.1073/pnas.88.21.9668 PMC52779

[3] C. C. Hanna , J. Kriegesmann , L. J. Dowman , C. F. W. Becker , R. J. Payne , Angew. Chemie, Int. Ed. 2022, 61, e202111266. 10.1002/anie.202111266 PMC9303669 34611966

[4] S. L. Campbell , M. R. Philips , Curr. Opin. Struct. Biol. 2021, 71, 180. 34365229 10.1016/j.sbi.2021.06.015 PMC8649064

[5] K. T. Lane , L. S. Beese , J. Lipid Res. 2006, 47, 681. 16477080 10.1194/jlr.R600002-JLR200

[6] S. E. Hampton , T. M. Dore , W. K. Schmidt , Crit. Rev. Biochem. Mol. Biol. 2018, 53, 157. 29424242 10.1080/10409238.2018.1431606 PMC5874806

[7] S. Kuchay , H. Wang , A. Marzio , K. Jain , H. Homer , N. Fehrenbacher , M. R. Philips , N. Zheng , M. Pagano , Nat. Struct. Mol. Biol. 2019, 26, 628. 31209342 10.1038/s41594-019-0249-3 PMC6609460

[8] Y. Zhang , Y. Goto , H. Suga , Trends Biochem. Sci. 2023, 48, 360. 36564250 10.1016/j.tibs.2022.11.002

[9] Y. Zheng , Y. Cong , E. W. Schmidt , S. K. Nair , Acc. Chem. Res. 2022, 55, 1313. 35442036 10.1021/acs.accounts.2c00108 PMC9519032

[10] M. Montalbán‐López , T. A. Scott , S. Ramesh , I. R. Rahman , A. J. van Heel , J. H. Viel , V. Bandarian , E. Dittmann , O. Genilloud , Y. Goto , M. J. Grande Burgos , C. Hill , S. Kim , J. Koehnke , J. A. Latham , A. J. Link , B. Martínez , S. K. Nair , Y. Nicolet , S. Rebuffat , H.‐G. Sahl , D. Sareen , E. W. Schmidt , L. Schmitt , K. Severinov , R. D. Süssmuth , A. W. Truman , H. Wang , J.‐K. Weng , G. P. van Wezel , et al., Nat. Prod. Rep. 2021, 38, 130. 32935693 10.1039/d0np00027b PMC7864896

[11] M. Purushothaman , S. Sarkar , M. Morita , M. Gugger , E. W. Schmidt , B. I. Morinaka , Angew. Chemie, Int. Ed. 2021, 60, 8460. 10.1002/anie.202015975 PMC8011950 33586286

[12] M. D. Tianero , E. Pierce , S. Raghuraman , D. Sardar , J. A. Mcintosh , J. R. Heemstra , Z. Schonrock , B. C. Covington , J. A. Maschek , J. E. Cox , B. O. Bachmann , B. M. Olivera , D. E. Ruffner , E. W. Schmidt , Proc. Natl. Acad. Sci. USA 2016, 113, 1772. 26831074 10.1073/pnas.1525438113 PMC4763782

[13] J. A. Mcintosh , M. S. Donia , S. K. Nair , E. W. Schmidt , J. Am. Chem. Soc. 2011, 133, 13698. 21766822 10.1021/ja205458h PMC3170831

[14] Y. Hao , E. Pierce , D. Roe , M. Morita , J. A. Mcintosh , V. Agarwal , T. E. Cheatham , E. W. Schmidt , S. K. Nair , Proc. Natl. Acad. Sci. USA 2016, 113, 14037. 27872314 10.1073/pnas.1609869113 PMC5150373

[15] M. Morita , Y. Hao , J. K. Jokela , D. Sardar , Z. Lin , K. Sivonen , S. K. Nair , E. W. Schmidt , J. Am. Chem. Soc. 2018, 140, 6044. 29701961 10.1021/jacs.8b03137 PMC6242345

[16] J. Martins , N. Leikoski , M. Wahlsten , J. Azevedo , J. Antunes , J. Jokela , K. Sivonen , V. Vasconcelos , D. P. Fewer , P. N. Leão , Sci. Rep. 2018, 8, 14537. 30266955 10.1038/s41598-018-32618-5 PMC6162287

[17] L. Dalponte , A. Parajuli , E. Younger , A. Mattila , J. Jokela , M. Wahlsten , N. Leikoski , K. Sivonen , S. A. Jarmusch , W. E. Houssen , D. P. Fewer , Biochemistry 2018, 57, 6860. 30452235 10.1021/acs.biochem.8b00879

[18] Y. Zhang , K. Hamada , D. T. Nguyen , S. Inoue , M. Satake , S. Kobayashi , C. Okada , K. Ogata , M. Okada , T. Sengoku , Y. Goto , H. Suga , Nat. Catal. 2022, 5, 682.

[19] C. S. Phan , K. Matsuda , N. Balloo , K. Fujita , T. Wakimoto , T. Okino , J. Am. Chem. Soc. 2021, 143, 10083. 34181406 10.1021/jacs.1c05732

[20] A. Mattila , R.‐M. Andsten , M. Jumppanen , M. Assante , J. Jokela , M. Wahlsten , K. M. Mikula , C. Sigindere , D. H. Kwak , M. Gugger , H. Koskela , K. Sivonen , X. Liu , J. Yli‐Kauhaluoma , H. Iwaï , D. P. Fewer , ACS Chem. Biol. 2019, 14, 2683. 31674754 10.1021/acschembio.9b00620

[21] N. Leikoski , L. Liu , J. Jokela , M. Wahlsten , M. Gugger , A. Calteau , P. Permi , C. A. Kerfeld , K. Sivonen , D. P. Fewer , Chem. Biol. 2013, 20, 1033. 23911585 10.1016/j.chembiol.2013.06.015

[22] D. Sardar , Y. Hao , Z. Lin , M. Morita , S. K. Nair , E. W. Schmidt , J. Am. Chem. Soc. 2017, 139, 2884. 28195477 10.1021/jacs.6b12872 PMC5764894

[23] M. Okada , I. Sato , S. J. Cho , H. Iwata , T. Nishio , D. Dubnau , Y. Sakagami , Nat. Chem. Biol. 2005, 1, 23. 16407988 10.1038/nchembio709

[24] M. Okada , S. Sumimoto , ACS Symp. Ser. 2020, 1374, 201.

[25] K. Hirooka , S. Shioda , M. Okada , Biosci. Biotechnol. Biochem. 2020, 84, 347. 31670609 10.1080/09168451.2019.1685371

[26] M. Okada , A. Ishihara , R. Yamasaki , F. Tsuji , S. Hayashi , S. Usami , Y. Sakagami , Biosci. Biotechnol. Biochem. 2014, 78, 550. 25036949 10.1080/09168451.2014.891932

[27] B. Wang , H. Cheng , W. Qian , W. Zhao , C. Liang , C. Liu , G. Cui , H. Liu , L. Zhang , Genes Genet. Syst. 2020, 95, 141. 32611933 10.1266/ggs.19-00053

[28] E. N. Grady , J. Macdonald , L. Liu , A. Richman , Z.‐C. Yuan , Microb. Cell Fact. 2016, 15, 203. 27905924 10.1186/s12934-016-0603-7 PMC5134293

[29] K. Blin , S. Shaw , H. E. Augustijn , Z. L. Reitz , F. Biermann , M. Alanjary , A. Fetter , B. R. Terlouw , W. W. Metcalf , E. J. N. Helfrich , G. P. Van Wezel , M. H. Medema , T. Weber , Nucleic Acids Res. 2023, 51, W46. 37140036 10.1093/nar/gkad344 PMC10320115

[30] H. Ashkenazy , S. Abadi , E. Martz , O. Chay , I. Mayrose , T. Pupko , N. Ben‐Tal , Nucleic Acids Res. 2016, 44, W344. 27166375 10.1093/nar/gkw408 PMC4987940

[31] D. H. Haft , M. K. Basu , D. A. Mitchell , BMC Biol. 2010, 8, 70. 20500830 10.1186/1741-7007-8-70 PMC2887384

[32] P. Shannon , A. Markiel , O. Ozier , N. S. Baliga , J. T. Wang , D. Ramage , N. Amin , B. Schwikowski , T. Ideker , Genome Res. 2003, 13, 2498. 14597658 10.1101/gr.1239303 PMC403769

[33] R. Zallot , N. Oberg , J. A. Gerlt , Biochemistry 2019, 58, 4169. 31553576 10.1021/acs.biochem.9b00735 PMC7057060

[34] J. Jumper , R. Evans , A. Pritzel , T. Green , M. Figurnov , O. Ronneberger , K. Tunyasuvunakool , R. Bates , A. Z Dek , A. Potapenko , A. Bridgland , C. Meyer , S. A. A. Kohl , A. J. Ballard , A. Cowie , B. Romera‐Paredes , S. Nikolov , R. Jain , J. Adler , T. Back , S. Petersen , D. Reiman , E. Clancy , M. Zielinski , M. Steinegger , M. Pacholska , T. Berghammer , S. Bodenstein , D. Silver , O. Vinyals , et al., Nature 2021, 596, 583. 34265844 10.1038/s41586-021-03819-2 PMC8371605

[35] M. Mirdita , K. Schütze , Y. Moriwaki , L. Heo , S. Ovchinnikov , M. Steinegger , Nat. Methods 2022, 19, 679. 35637307 10.1038/s41592-022-01488-1 PMC9184281

[36] Y. Shen , A. Bax , J. Biomol. NMR 2010, 46, 199. 20041279 10.1007/s10858-009-9395-y PMC2847849

[37] L. Y. P. Luk , M. E. Tanner , J. Am. Chem. Soc. 2009, 131, 13932. 19743851 10.1021/ja906485u

[38] F. Ecker , A. Vattekkatte , W. Boland , M. Groll , Nat. Chem. 2023, 15, 1188. 37308711 10.1038/s41557-023-01235-9 PMC10396970

[39] J. Jung , T. Mori , C. Kobayashi , Y. Matsunaga , T. Yoda , M. Feig , Y. Sugita , Wiley Interdiscip. Rev. Comput. Mol. Sci. 2015, 5, 310. 26753008 10.1002/wcms.1220 PMC4696414

[40] F. H. Wallrapp , J.‐J. Pan , G. Ramamoorthy , D. E. Almonacid , B. S. Hillerich , R. Seidel , Y. Patskovsky , P. C. Babbitt , S. C. Almo , M. P. Jacobson , C. D. Poulter , Proc. Natl. Acad. Sci. USA 2013, 110, E1196. 23493556 10.1073/pnas.1300632110 PMC3612614

[41] J. Konc , D. Janezic , Nucleic Acids Res. 2014, 42, W215. 24861616 10.1093/nar/gku460 PMC4086080

[42] M. E. Tanner , Nat. Prod. Rep. 2015, 32, 88. 25270661 10.1039/c4np00099d

[43] F. Tsuji , A. Ishihara , A. Nakagawa , M. Okada , S. Kitamura , K. Kanamaru , Y. Masuda , K. Murakami , K. Irie , Y. Sakagami , Biosci. Biotechnol. Biochem. 2012, 76, 1492. 22878193 10.1271/bbb.120206

[44] M. Okada , H. Yamaguchi , I. Sato , F. Tsuji , D. Dubnau , Y. Sakagami , Biosci. Biotechnol. Biochem. 2008, 72, 914. 18323630 10.1271/bbb.80006

