# Supplementary material for: Structure Prediction and Genome Mining‐Aided Discovery of the Bacterial C‐Terminal Tryptophan Prenyltransferase PalQ
Source: Adv Sci (Weinh). 2023 Dec 7;11(6):2307372. doi: 10.1002/advs.202307372 (PMC10853753; doi:10.1002/advs.202307372)
Supplement: Supplementary file 1 — Supporting Information [file ADVS-11-2307372-s001.pdf]

ADVANCED SCIENCE
Open Access

Supporting Information

for *Adv. Sci.*, DOI 10.1002/advs.202307372

Structure Prediction and Genome Mining-Aided Discovery of the Bacterial C-Terminal Tryptophan Prenyltransferase PalQ

Azusa Miyata, Sohei Ito and Daisuke Fujinami**

Structure Prediction and Genome Mining-Aided Discovery of the Bacterial C-terminal Tryptophan Prenyltransferase PalQ.

Azusa Miyata, Sohei Ito* and Daisuke Fujinami*

Graduate Division of Nutritional and Environmental Sciences, University of Shizuoka, 52-1 Yada, Suruga-ku, Shizuoka 422-8526, Japan

Section S1: Materials and methods

Bioinformatic analysis

Automated identification of the PalX cluster was performed with antiSMASH 7.0[1], using the *Paenibacillus alvei* genome (Genome ID: AMBZ01000001). Sequence similarity networks were generated with the Enzyme Function Initiative-Enzyme Similarity Tool (EFI-ESI) available online[2]. The isoprene synthase domain superfamily (InterPro: IPR008949) was utilized as the query. Nodes were clustered based on an alignment score threshold of 10^{-30}. The resultant network was visualized using Cytoscape 3.7.1[3].

Cloning of palX and palQ genes

All primers used for cloning and mutagenesis are detailed in Table S2. The strain *Paenibacillus alvei* JCM 20131/ATCC 6344 was purchased from RIKEN BRC. The *palX* and *palQ* genes were amplified from *P. alvei* cells using PrimeSTAR GXL DNA Polymerase (TAKARA), and then cloned into the pET28b vector at the SalI and NheI sites, using an In-fusion HD cloning kit (Clontech). The D200A/D204A double mutant was constructed using KOD-mutagenesis kit (TOYOBO). Turbo Competent *E. coli* (NEB) was chosen as the host for plasmid preparation. The successful DNA cloning was confirmed by DNA sequencing using T7 promoter and T7 terminator primers.

Overexpression and purification of palX and palQ

The plasmids were transformed into the BL21(DE3) *E. coli* strain. BL21(DE3) cells harboring the relevant plasmids were cultured in LB broth supplemented with 30 µg/mL of kanamycin at 37 °C. At an OD_{600} between 0.6 and 0.8, the expression was induced by adding IPTG to a final concentration of 0.5 mM and subsequently incubating the culture at 23 °C for 16 hours. Cells were harvested by centrifugation, and the cell pellet was resuspended in buffer A, consisting of 50 mM Tris-HCl (pH 9.0), 150 mM NaCl, and 10% glycerol. The cell suspension was sonicated, and the lysate was centrifuged at 11,000 g for 30 minutes to remove cell debris. The supernatant, containing the overexpressed proteins, was loaded onto a HisTrap HP column (Cytiva) pre-equilibrated with buffer A. The column was washed thoroughly with buffer A containing 20 mM imidazole, after which the bound proteins were eluted using buffer A containing 100 mM and 400 mM imidazole. To prevent PalQ precipitation, Gly-KOH (pH 10.0) and TCEP were added to the elution fraction at final concentrations of 100 mM and 1 mM, respectively. The concentrations of PalQ and PalX were determined by measuring the absorbance at 280 nm. His-tag purified PalQ and PalX were further subjected to size exclusion chromatography (SEC) using a Superdex 200 increase column (Cytiva). The running buffer consisted of 20 mM Gly-KOH (pH 10.0), 150 mM NaCl, 5% glycerol, and 1 mM TCEP. Eluted protein fractions were subsequently analyzed by SDS-PAGE. To confirm the formation of the PalX-PalQ complex, SEC was performed on a 1:1 mixture

of the samples under the same conditions. Native agarose gel electrophoresis was carried out using a 1.7% agarose gel, in buffer composed of 25 mM Tris and 190 mM glycine (pH 8.3).

End point prenylation assay

The reaction mixture for the donor substrate assay contained 100 μM PalX with 10 μM PalQ, 5 mM $MgCl_2$, and 200 μM of prenyl diphosphates: either DMAPP ammonium salts (Cayman Chemical), GPP ammonium salts (Axon Medchem), FPP ammonium salts (Axon Medchem), or GGPP ammonium salts (Cayman Chemical). The reaction mixture for the acceptor substrate assay consisted of 10 μM PalQ, 200 μM DMAPP, 5 mM $MgCl_2$, and 100 μM of various core peptides, which were chemically synthesized and sourced from Genscript. The reaction mixture for the metal-dependence assay included 100 μM DNVRRFFW, 10 μM PalQ, 5 mM each metal, and 200 μM DMAPP. The reaction mixture for the PalQ mutation assay included 100 μM PalX, 15 μM PalQ WT or PalQ D200A/D204A, 5 mM each metal, and 400 μM DMAPP. All reaction mixtures were buffered by 50 mM Tris-HCl, pH 9.0, 150 mM NaCl, 1 mM TCEP, and 10% glycerol, incubated for 18 hours at room temperature (RT), and then desalted using a C18 ZipTip (Merck Millipore) according to the manufacturer's protocol. One µl of sample solution was mixed with 1 µl of matrix solution (15 mg/mL 2,5-dihydroxybenzoic acid in acetonitrile) on the target plate and analyzed by MALDI-TOF-MS (Bruker).

Kinetic analysis

The assay solution consisted of 50 mM Tris-HCl, pH 9.0, 150 mM NaCl, 1 mM TCEP, 10% glycerol, 5 mM $MgCl_2$, and a nominal amount of 10 nM PalQ. For the donor substrate kinetics, the reaction mixture was formulated with a range of concentrations (0.01 to 2.5 mM) of the donor substrates; specifically DMAPP, GPP, FPP. This mixture also contained 200 μM of PalX. The mixture designated for acceptor substrate kinetics was composed of 2 mM DMAPP and either PalX at concentrations between 0.005 and 0.25 mM or DNVRRFFW ranging from 0.01 to 2.5 mM. The mixtures were incubated for 4 hours at RT. For the kinetics of DNVRRFFW, the incubation time was changed to 18 hours because of its low reactivity. The reactions were stopped by the addition of EDTA (pH 8.0) to a final concentration of 100 mM. Precipitated protein was removed by centrifugation before the analysis of the reactions by HPLC (Shimadzu).

The HPLC conditions were as follows: the mobile phase consisted of (A) H_2O containing 0.1% TFA and (B) acetonitrile containing 0.1% TFA, in a gradient from 80:20 to 20:80 over 10 min. The flow rate was 1.00 mL/min. The column was COSMOSIL $_5C_{18}$-AR-II (4.6 mm × 100 mm, Nacalai Tesque). The column oven temperature was set at 40 °C and the injection volume was 50 μL. Quantification of both substrate and product was achieved by measuring the absorbance at 260 nm. Kinetic parameters were estimated by fitting the initial velocities to the Michaelis-Menten equation (for DMAPP) or the substrate inhibition equation (for GPP, FPP, NHLP and DNVRRFFW) by a nonlinear least squares method, using the ORIGIN software (OriginLab).

Prenylation assay in preparative scale

The 3 ml assay solution contained 200 μM DNVRRFFW, 400 μM DMAPP, 2.5 mM $MgCl_2$ and 10 μM of PalQ, dissolved in 5 mM Tris-HCl, pH 9.0, 15 mM NaCl, 40 mM imidazole, 0.1 mM TCEP, and 2.5 mM $MgCl_2$. The reaction mixtures were incubated overnight at RT. The HPLC conditions were as follows: the mobile phase consisted of (A) 20 mM ammonium formic acid, pH 7.5, and (B) 80% acetonitrile with 20 mM ammonium formic acid, pH 7.5, in a gradient from

100:0 to 100:0 over 30 min. The flow rate was 1.00 mL/min. The column was COSMOSIL $_5C_{18}$-AR-II (4.6 mm × 100 mm, Nacalai Tesque). The column oven temperature was set at 40 °C and the injection volume was 1,500 µL. The elution fraction containing prenylated DNVRRFFW was dried to powder in a centrifugal concentrator.

NMR

The 0.2 mL NMR sample contained 0.9 mM prenylated DNVRRFFW, dissolved in 100% ^2H_2O. NMR spectra were recorded on a Bruker Avance600 spectrometer equipped with a TXI cryoprobe, at 298 K. ^1H and ^{13}C resonance assignments were performed using ^1H-^1H DQF-COSY, ^1H-^1H TOCSY (mixing time 45 ms), ^1H-^1H ROESY (mixing time 225 ms), ^1H-^{13}C gHSQC, and ^1H-^{13}C HMBC. The backbone ^1HN assignments were conducted based on the ^1H-^1H COSY spectrum of 0.9 mM prenylated DNVRRFFW dissolved in 10% ^2H_2O. NMR data were processed and displayed with the program nmrPipe/nmrDraw, version 3.0[4]. The ^1H and ^{13}C resonance assignments are provided in Table S3.

Molecular modeling

The multimeric structures of PalQ and the PalX complex was predicted by AlphaFold2 via ColabFold[5,6]. The magnesium ions were positioned based on the confidence score from the ProBiS server[7]. DMAPP (PDBID: DMA) and FPP (PDBID: FPP) were modeled individually using the "drag coordinates" function within the PyMOL Molecular Graphics System, version 2.3.5 (Schrödinger, LLC). For this modeling, we drew guidance from the X-ray structure of the bacterial polyprenyl synthase complex (PDBID: 3OYR)[8]. The selection of 3OYR as a reference was supported by the structural similarity, as indicated by the DALI server[9]. The CHARMM force field parameters for each isoprenyl diphosphate were created using CGenFF. The PDB files corresponding to each isoprenyl diphosphate were transformed to the mol2 format, and adjusted to pH 7 conditions with the Open Babel software[10]. These mol2 files served as input for CGenFF.

The constructed structures were then subjected to molecular dynamics simulations using GENESIS 1.6.0[11] These simulations were performed with the Generalized Born/Surface Area (GBSA) implicit solvation model. Production runs were performed for 10 ns at 298.15 K. All bonds inclusive of hydrogen atoms remained constrained during the production run. The time step for integration was set to 2 fs. Temperature and pressure were regulated by the Langevin thermostat. The MD trajectories were analyzed and visualized by VMD[12].

Molecular modeling of the complex consisting of *B. subtilis* strain 168 ComQ, ComX, FPP, and Mg^{2+} was conducted in a manner similar to that of the PalQ-PalX complex described above.

Section S2: Tables and Figures

Table S1

List of homologous enzymes and their putative precursors within the sequence similarity network cluster containing PalQ.

Bacterial species	Putative prenyltransferase	Putative acceptor peptide
Paenibacillus bovis	A0A172ZFF2	A0A172ZED2
Paenibacillus curdlanolyticus	E0IAM1	E0IAM2
Cohnella fermenti	A0A4S4C8C8	-
Paenibacillus baekrokdamisoli	A0A3G9ITL8	-
Paenibacillus pasadenensis	A0A2N5NCE9	A0A2N5NCF0
Clostridium sp	A0A7X7XZX4	A0A7X7XYV3
Cohnella abietis	A0A3T1DEA5	A0A3T1DE98
Geobacillus sp. (strain Y412MC10)	D3EJN1	D3EJN0
Paenibacillus chitinolyticus	A0A410X357	A0A410X384
Paenibacillaceae bacterium	A0A4Q2LZT4	A0A4Q2LY61
Paenibacillus sp. oral taxon 786 str. D14	C6J525	C6J524
Paenibacillus alvei	K4ZRG6 (PalQ)	K4ZQB1 (PalX)
Paenibacillus sp. FSL H7-0357	A0A089HZA4	A0A089J4Q2
Paenibacillus sp. Root444D2	A0A0Q7J549	A0A0Q7IQ32
Paenibacillus protaetiae	A0A4P6FBV7	A0A4P6F4D2

Table S2

List of primers.

Primer	Sequence	
PalX_f	GCAGCCATATGGCTAGCATGGTTTCTGCGGATCAA	This study
PalX_r	TGGTGGTGGTGCTCGAGTTCATGAGAATGATAGGG	This study
PalQ_f	GCAGCCATATGGCTAGCATGCATTGGTATGAACCG	This study
PalQ_r	TGGTGGTGGTGCTCGAGTCTACTTTGTTACCGAAT	This study
PalQ_D200A/D204A_f	GGATTGGGCAGCGTGGCAGACAG	This study
PalQ_D200A/D204A_r	GCATTCATTTGCAAACAGATAAGCGCAGT	This study
pET28b_SalI_f	ACTCGAGCACCACCACCACCACCACTGA	This study
pET28b_NheI_r	CGCGCGGCAGCCATATGGCTAGC	This study
T7P	TAATACGACTCACTATAGGG	This study
T7T	ATGCTAGTTATTGCTCAGCGG	This study

Table S3

Chemical shifts of prenylated DNVRRFFW.

Residue	Position	^{1}H	^{13}C
D1	CO		174.5
	N	n.d.	
	α	4.09	52.9
	β	2.66/2.71	40.8
	γ		177.8
N2	CO		173.4
	N	8.06	
	α	4.72	55.2
	β	2.68/2.76	38.3
	γ		176.7
V3	CO		175.6

	N	8.08	
	α	3.97	62.1
	β	1.99	32.2
	γ1	0.86	20.2
	γ1	0.82	20.5
R4 or R5	CO		174.9
	N	8.18	
	α	4.19	55.2
	β	1.55	30.6
	γ	1.4	26.6
	δ	3.04	40.7
	ζ		158.73
R4 or R5	CO		174.9
	N	8.31	
	α	4.19	55.2
	β	1.65	30.2
	γ	1.58	26.6
	δ	3.04	40.7
	ζ		158.73
F6	CO		174.6
	N	7.89	
	α	4.52	55.9
	β	2.71	41.2
	γ		137.7
	δ1/ δ2	6.92	131.4
	ε1/ ε2	6.97	130.8
	ζ	7.02	129.3
F7	CO		174.6
	N	7.89	
	α	4.62	55.2
	β	2.92/3.12	39.5
	γ		138.9
	δ1/ δ2	7.21	131.5
	ε1/ ε2	7.28	130.9
	ζ	7.21	129.3
F7’	CO		174.6
	N	8.15	
	α	4.91	55.3
	β	2.84/3.19	39.6
	γ		138.9
	δ1/ δ2	7.21	131.5
	ε1/ ε2	7.28	130.9
	ζ	7.21	129.3
W8	CO		180.8
	α	3.4	65.5
	β	2.14/2.48	42.3
	γ		57.7
	δ1		135.5
	δ2	5.27	84.5
	ε2		150.0
	ε3	7.12	131.0
	ζ2	6.78	113.2
	ζ3	6.86	122.6
	η3	7.14	126.0
	C1	2.30	37.1
	C2	5.04	120.9
	C3		138.7
	C4	1.37	19.3
	C5	1.58	27.2

W8’	CO		180.6
	α	3.88	65.2
	β	2.12/2.54	41.8
	γ		60.4
	δ1		135.3
	δ2	5.32	83.4
	ε2		149.7
	ε3	7.19	131.9
	ζ2	6.67	113.2
	ζ3	6.86	122.6
	η3	7.21	125.9
	C1	2.41	37.7
	C2	5.00	121.1
	C3		138.7
	C4	1.44	19.6
	C5	1.58	27.2

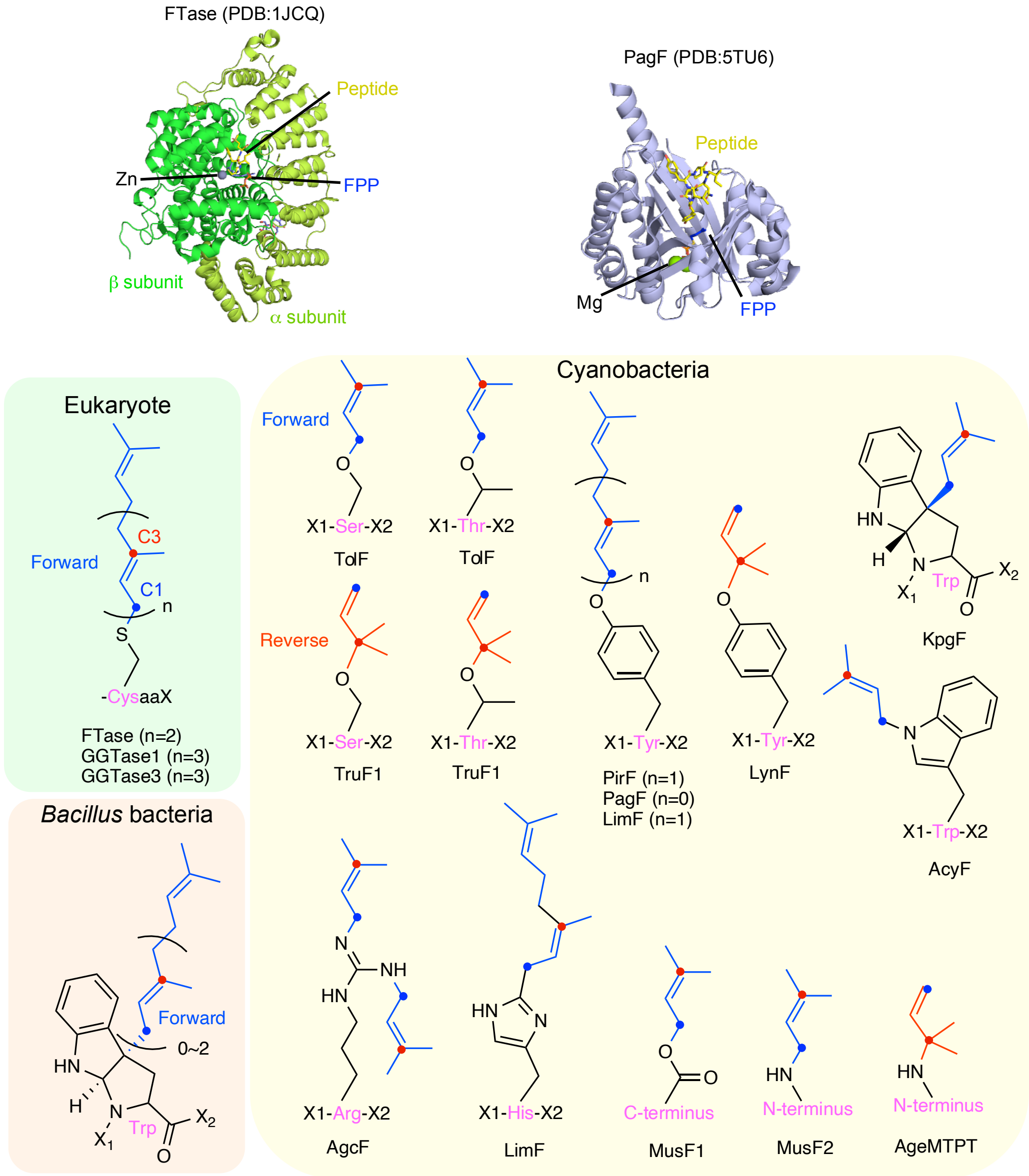

Figure S1. Chemical structures of eukaryotic and bacterial prenylations. Forward prenylations are represented by blue lines, while reverse prenylations are depicted in red. Prenylated residues are highlighted in magenta. X-ray structures of a representative eukaryotic prenyltransferase and a cyanobactin prenyltransferase are shown above.

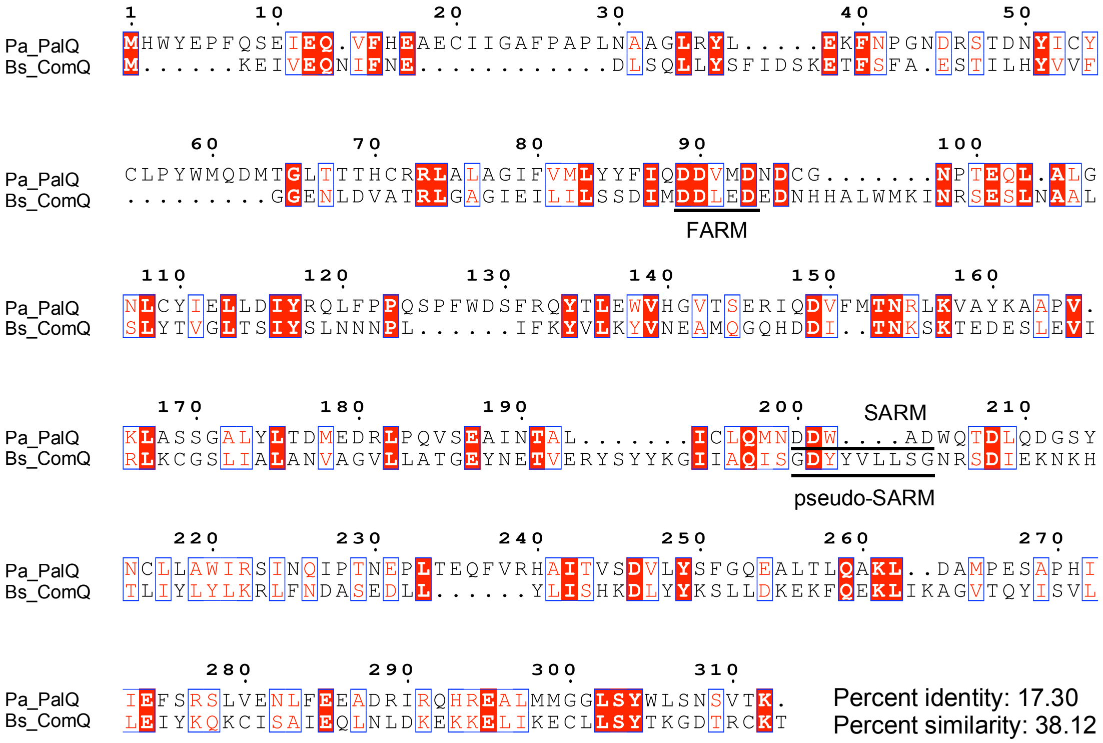

Figure S2. Sequence alignment of *Paenibacillus alvei* PalQ and *Bacillus subtilis* (strain 168) ComQ, visualized using ESPript 3.0[13]. Residues in the first aspartate-rich motif FARM and the (pseudo) second aspartate-rich motif SARM are underlined.

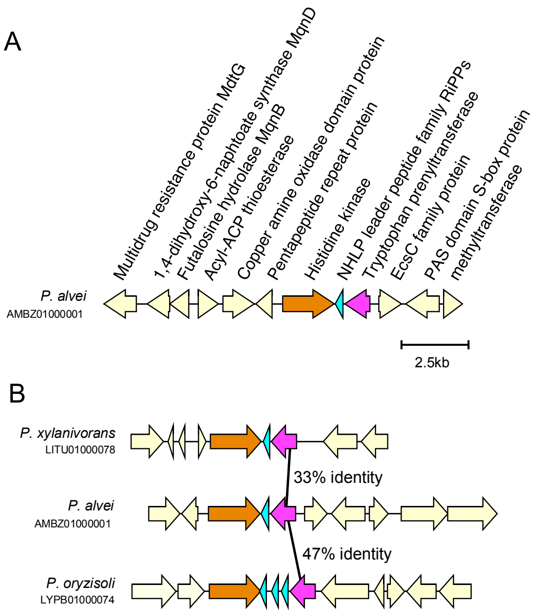

Figure S3. (A) Detailed representation of the *P. alvei* PalX biosynthetic gene cluster. The postulated functions of neighboring genes are indicated. (B) Gene alignment of the PalX cluster among *Paenibacillus xylanivorans*, *Paenibacillus alvei,* and *Paenibacillus oryzisoli*. Genome IDs for each bacterial species are displayed below their respective names.

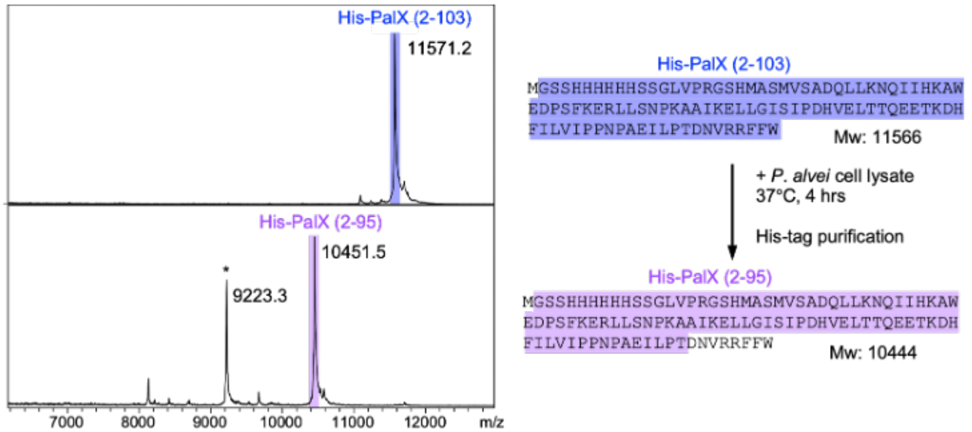

Figure S4. (A) MALDI-TOF-MS of His-tagged ComX incubated with and without the *Paenibacillus alvei* cell lysate. The peaks corresponding to His-PalX 2-103 and 2-95 are highlighted in blue and purple, respectively. Peaks indicated by an asterisk (*) could not be assigned based on amino acid sequence information.

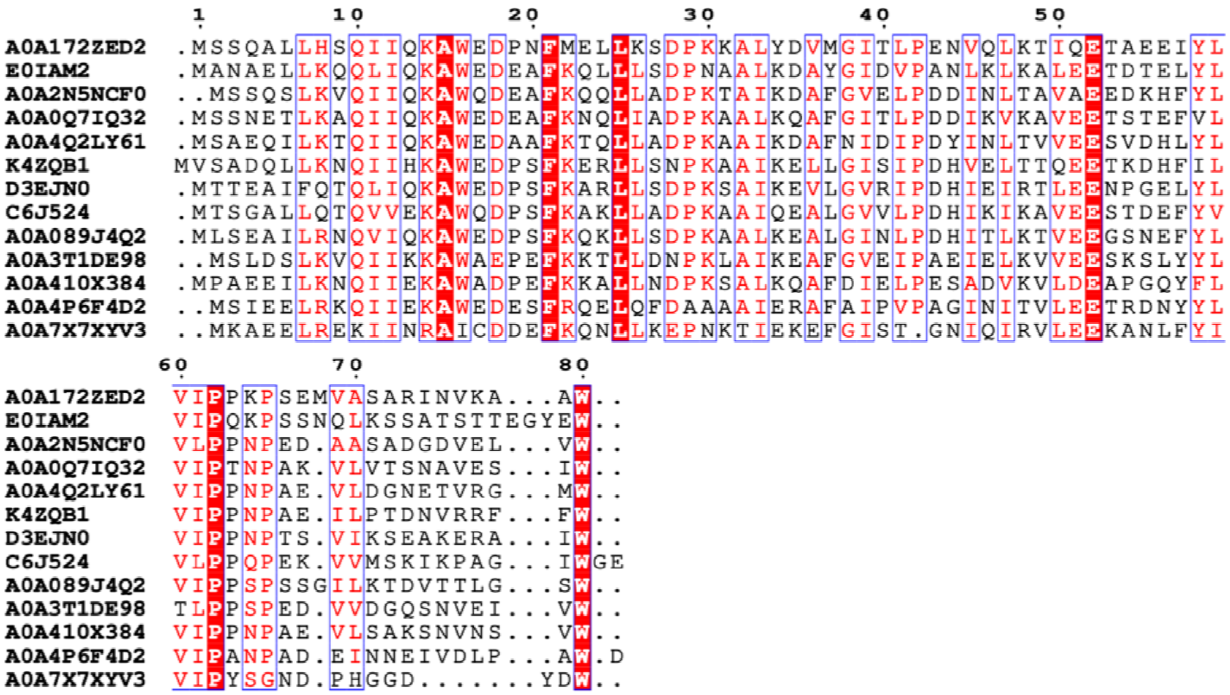

Figure S5. Sequence alignment of PalX homologues, as listed in Table S1.

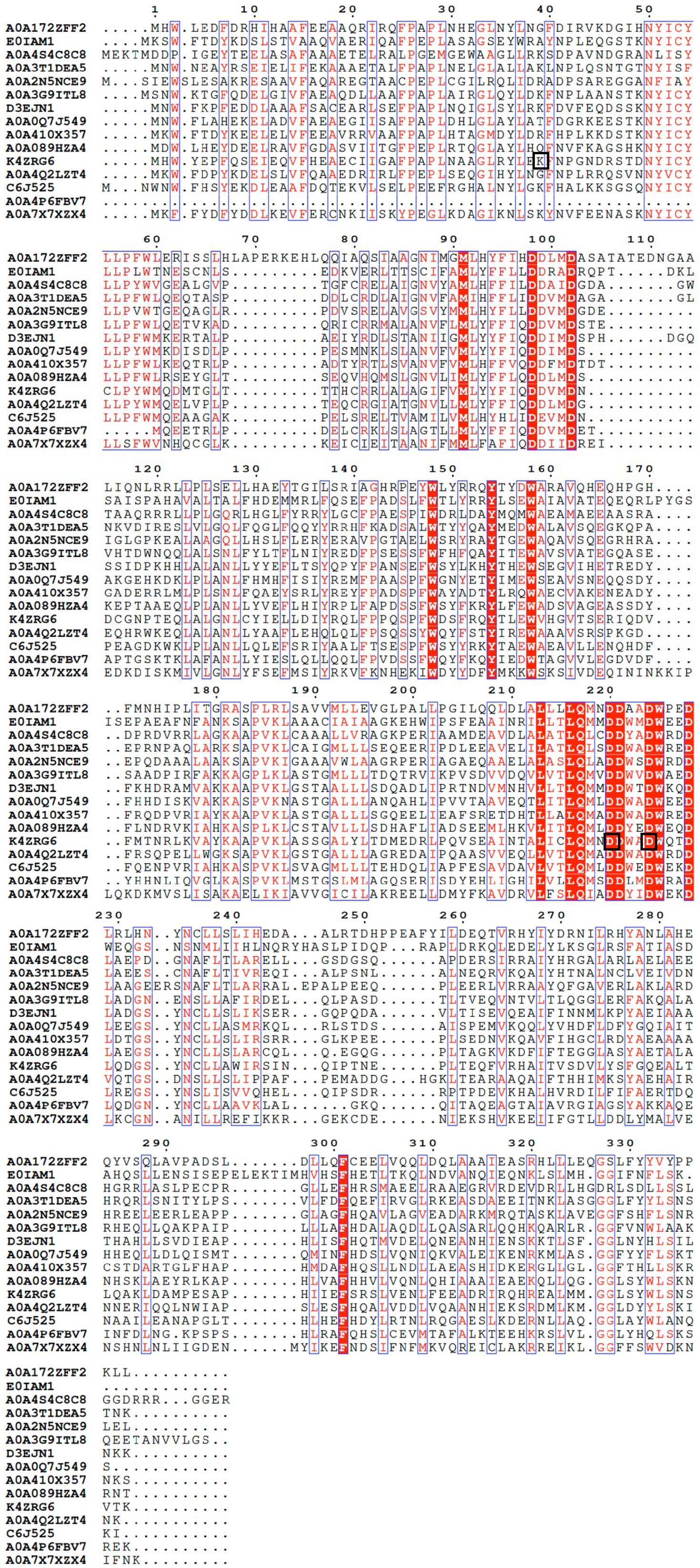

Figure S6. Sequence alignment of PalQ homologues, as listed in Table S1, with K39, D200, and D204 of *P. alvei* PalQ indicated by black boxes.

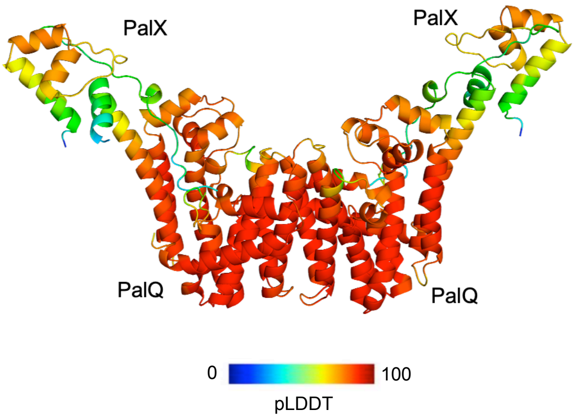

Figure S7. AlphaFold2 model of the PalQ and PalX complex, with the pLDDT confidence score depicted in red for high-confidence regions.

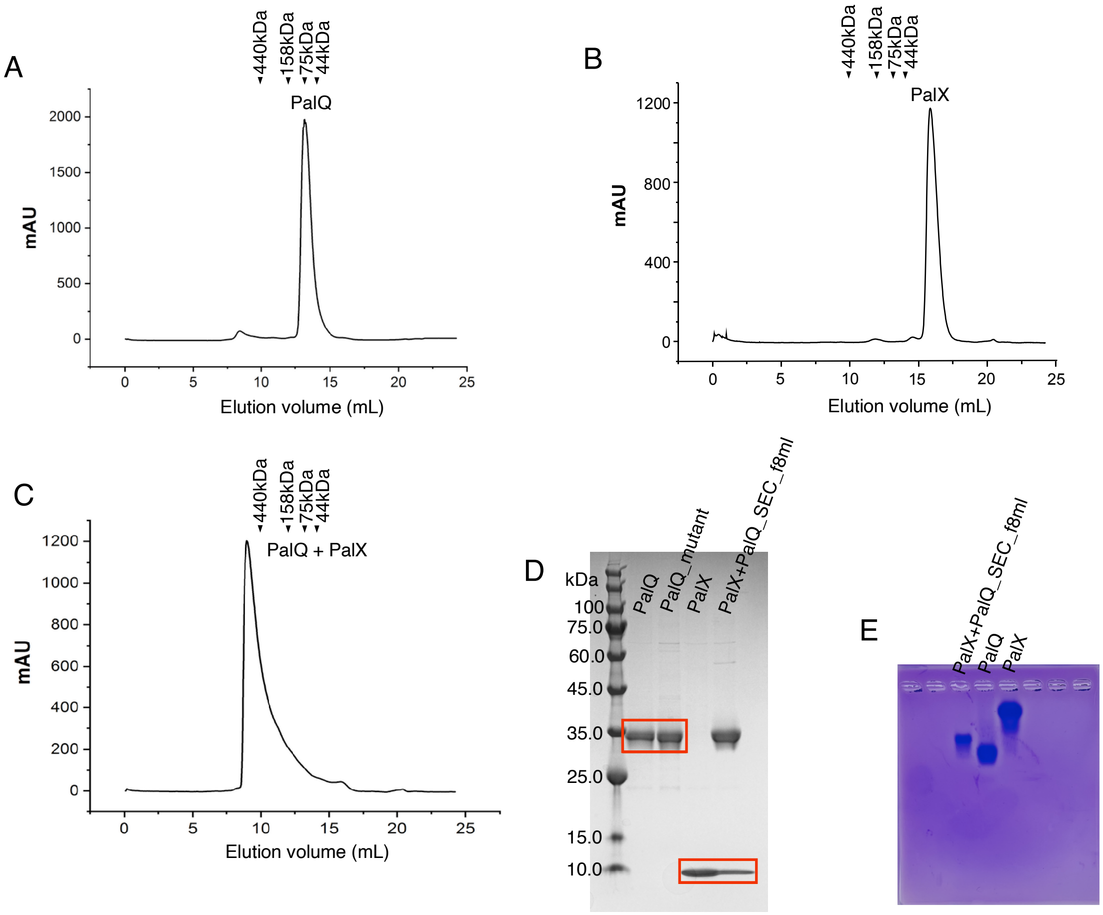

Figure S8. Size exclusion chromatography (SEC) analysis. (A) SEC analysis of PalQ following His-tag purification revealed an elution volume of 13.19 ml, which corresponds to a molecular weight of 76 kDa. This suggests the formation of a PalQ homodimer. (B) SEC analysis of PalX following His-tag purification. (C) A 1:1 molar mixture of PalX and PalQ was subjected to SEC. Both PalX and PalQ co-eluted in the void volume. (D) The protein fractions from the void volume

were further analyzed using SDS-PAGE. (E) In native agarose gel electrophoresis, the equimolar addition of PalX to PalQ induced band shifts, indicating the formation of a complex. ComX migrated more slowly than PalQ in native electrophoresis despite its lower molecular weight, likely due to the difference in pI (theoretical pIs: PalX, 6.53; PalQ, 5.09).

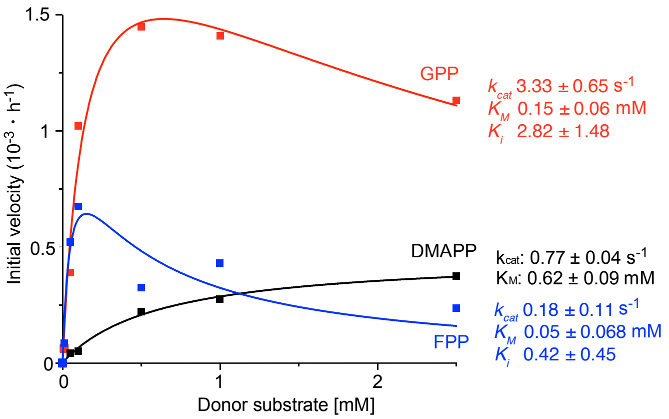

Figure S9. PalQ activity dependence on donor prenyl diphosphate concentrations. Data were derived from HPLC peak intensities at saturating levels of the acceptor, His-PalX. Kinetic parameters are also provided. For better fitting, we used a substrate inhibition model for GPP and FPP.

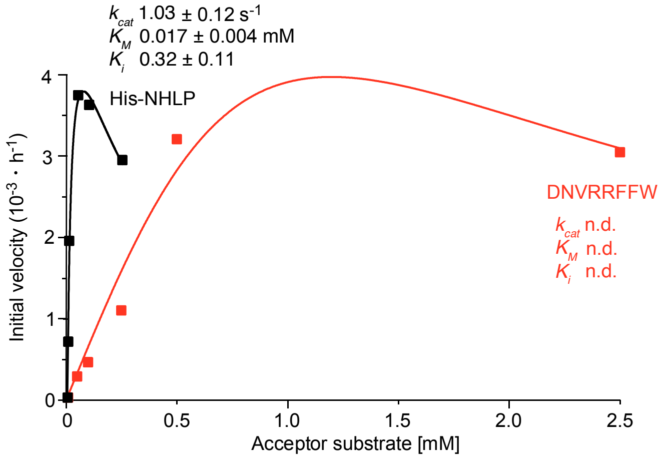

Figure S10. Dependence of PalQ activity on the concentrations of acceptor substrates. Data were determined using HPLC peak intensities at saturating concentrations of DMAPP. For better fitting, we used a substrate inhibition model. Data are shown as mean ± standard error of the mean.

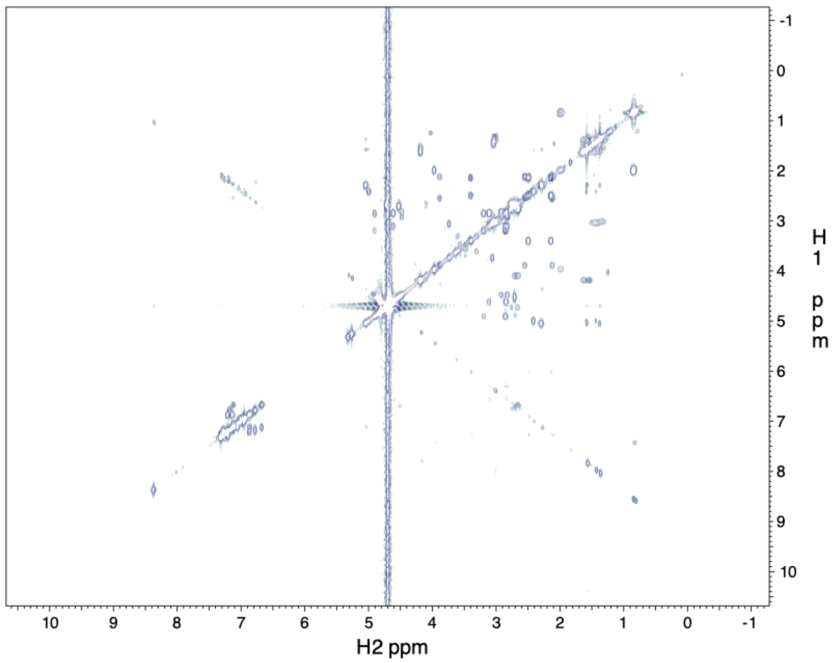

Figure S11. ^1H-^1H COSY spectrum of 0.9 mM prenylated DNVRRFFW in 100% ^2H_2O.

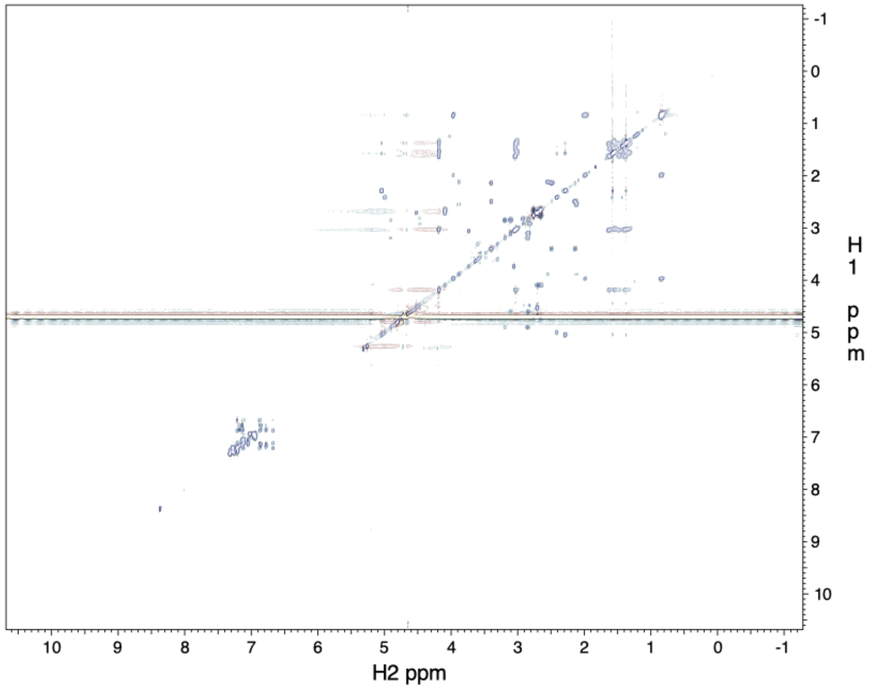

Figure S12. ^1H-^1H TOCSY spectrum of 0.9 mM prenylated DNVRRFFW in 100% ^2H_2O.

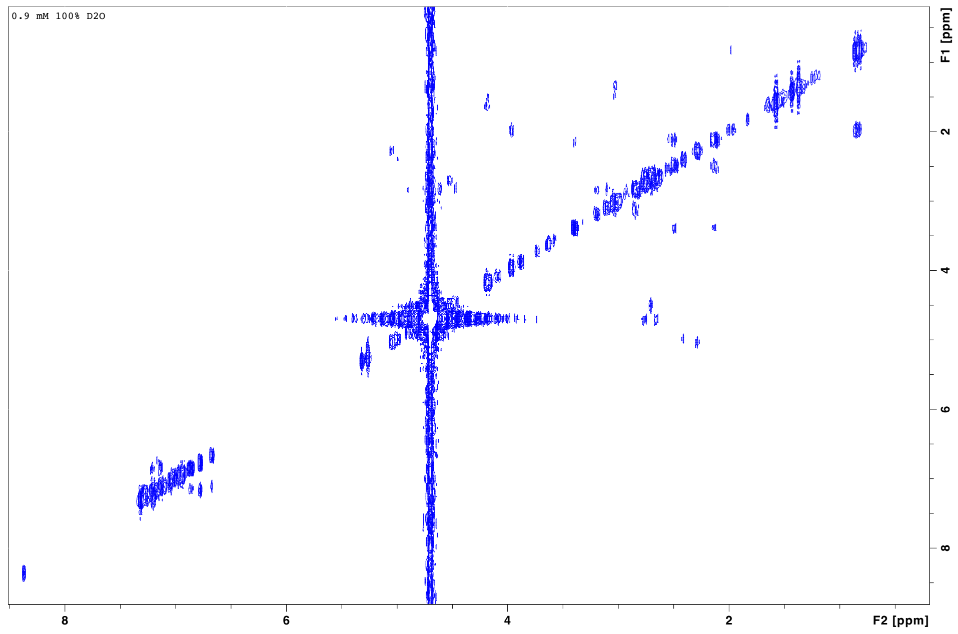

Figure S13. ^1H-^1H ROESY spectrum of 0.9 mM prenylated DNVRRFFW in 100% ^2H_2O.

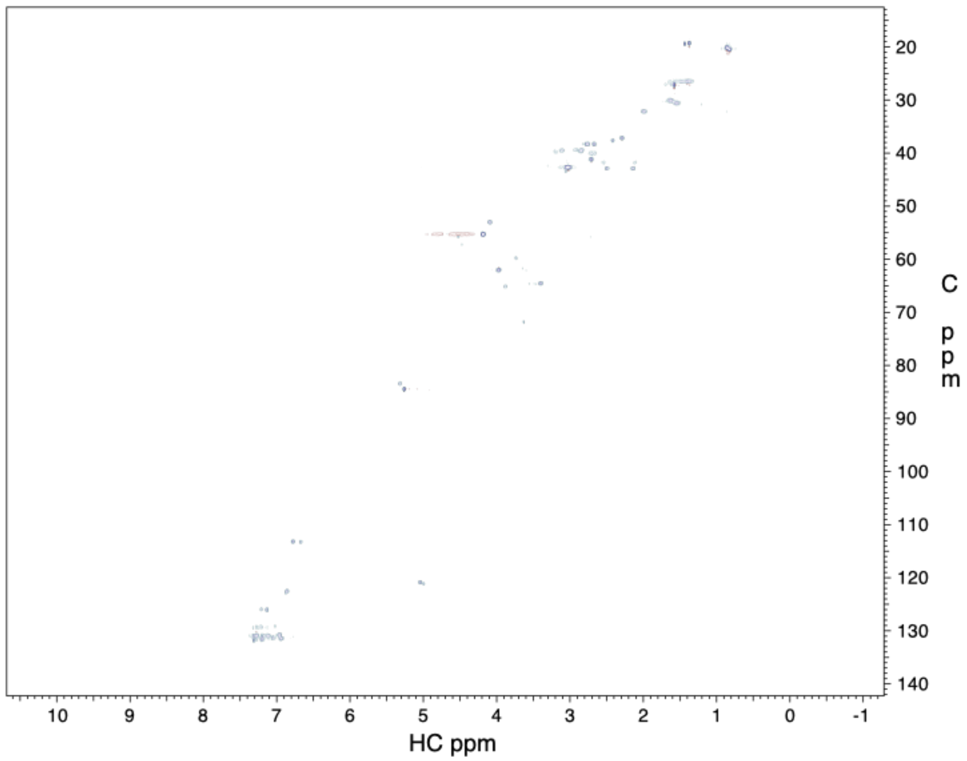

Figure S14. ^1H-^{13}C HSQC spectrum of 0.9 mM prenylated DNVRRFFW in 100% ^2H_2O.

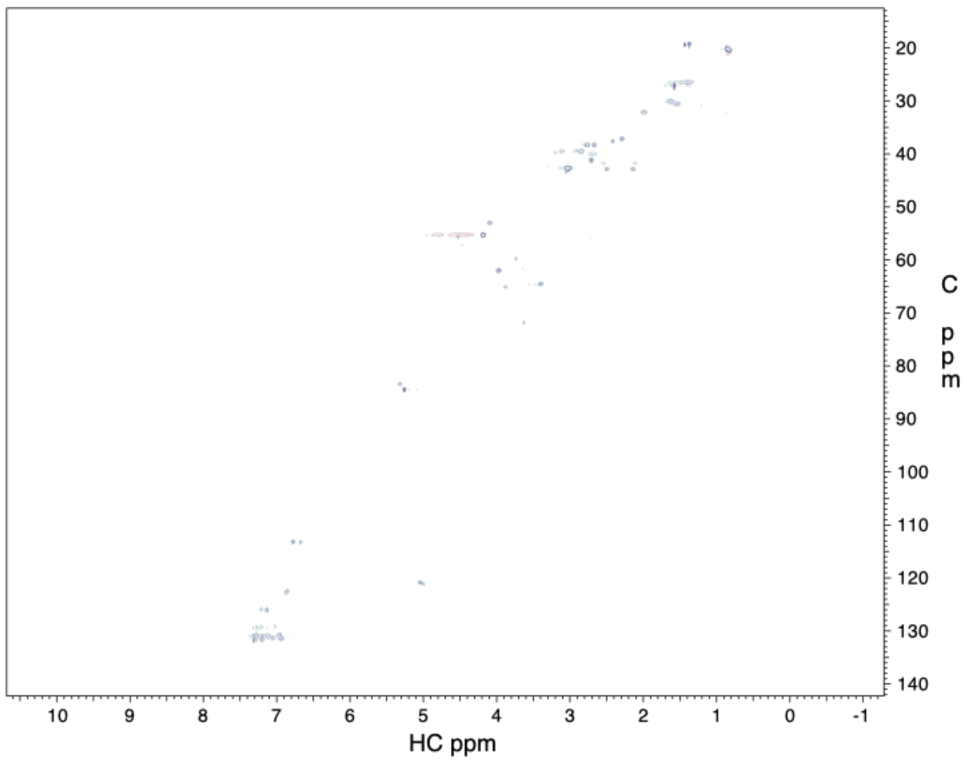

Figure S15. ^1H-^{13}C HMBC spectrum of 0.9 mM prenylated DNVRRFFW in 100% ^2H_2O.

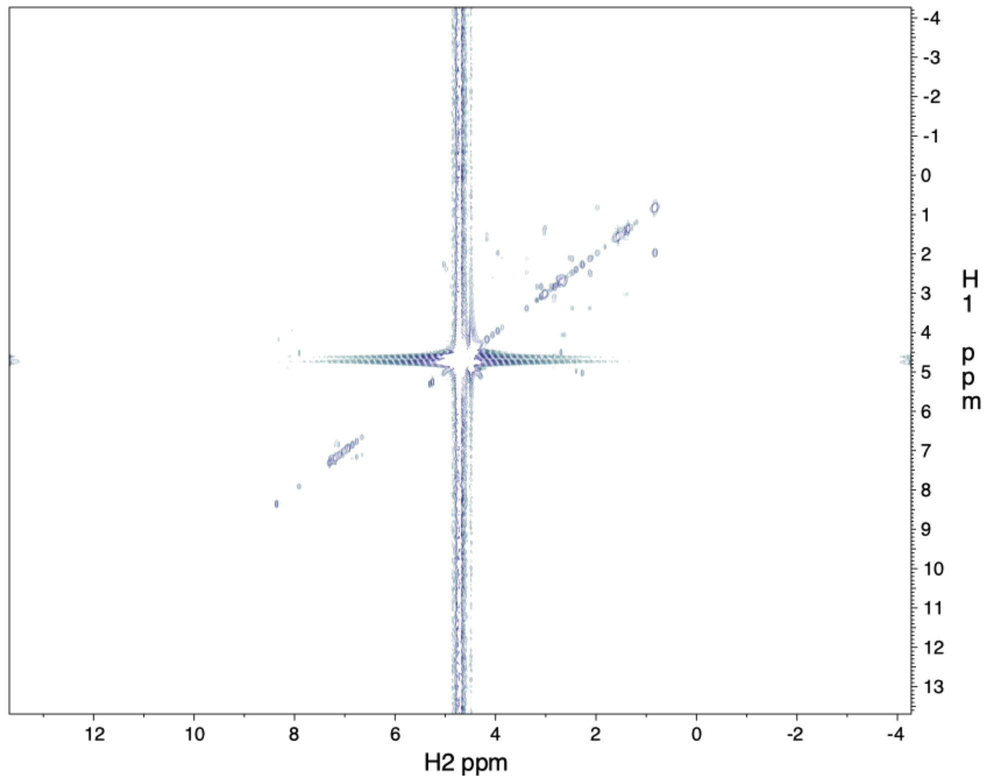

Figure S16. ^1H-^1H COSY spectrum of 0.9 mM prenylated DNVRRFFW in 10% ^2H_2O.

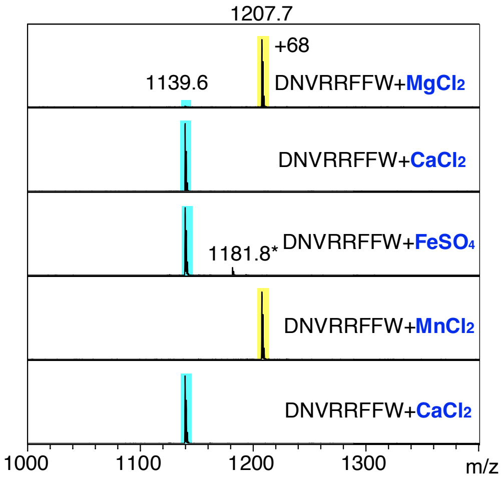

Figure S17. MALDI-TOF-MS spectra of DNVRRFFW after incubations with DMAPP, PalQ and various metal ions. Data were acquired at the endpoints of the prenylation assay. Peaks corresponding to unmodified and mono-prenylated peptides are highlighted in cyan and yellow, respectively.

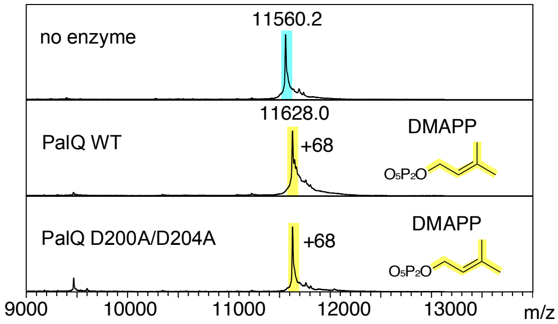

Figure S18. MALDI-TOF-MS spectra of His-tagged PalX following incubation with DMAPP in the presence of either wild-type PalQ or the D200A/D204A mutant.

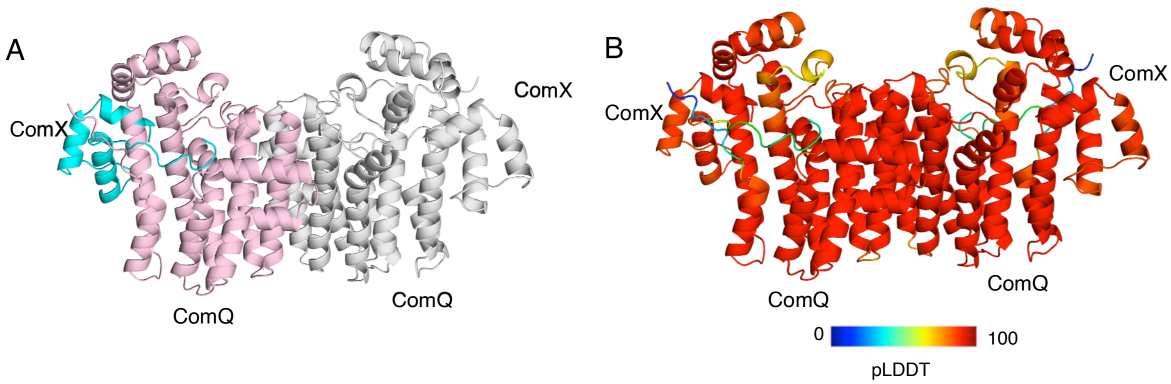

Figure S19. (A) AlphaFold2 model of the ComQ and ComX complex, (B) with the pLDDT confidence scores depicted in red to indicate high-confidence regions.

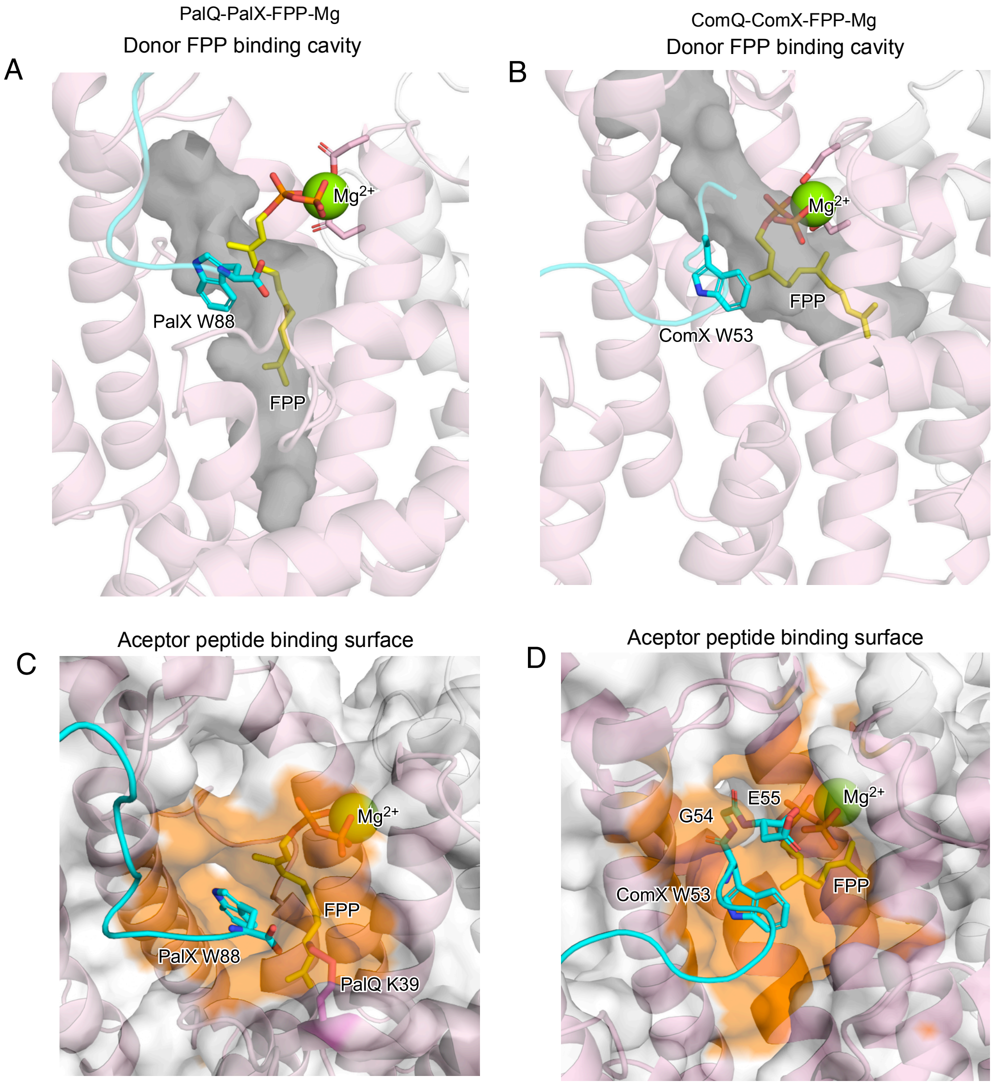

Figure S20. Structures of the active sites of PalQ and ComQ, obtained through MD simulations. Close-up views of the donor FPP binding cavities for PalQ (A) and ComQ (B) are shown with gray surface representations. Close-up views of the acceptor binding surfaces of PalQ (C) and ComQ (D), with the areas within 4.5 Å of the prenylated tryptophan highlighted in orange.

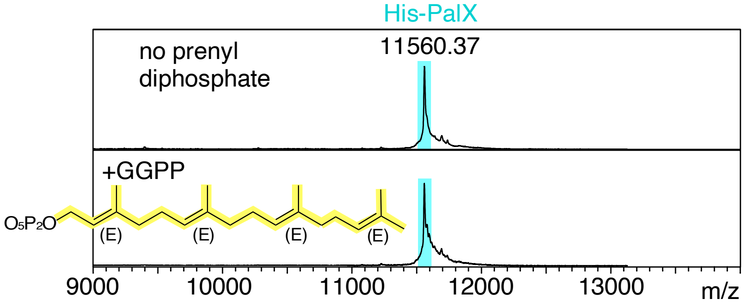

Figure S21. MALDI-TOF-MS spectra of His-PalX after incubations with GGPP and PalQ.

References

[1] K. Blin, S. Shaw, H. E. Augustijn, Z. L. Reitz, F. Biermann, M. Alanjary, A. Fetter, B. R. Terlouw, W. W. Metcalf, E. J. N. Helfrich, G. P. van Wezel, M. H. Medema, T. Weber, *Nucleic Acids Res.* **2023**, *51*, 46–50.

[2] J. A. Gerlt, J. T. Bouvier, D. B. Davidson, H. J. Imker, B. Sadkhin, D. R. Slater, K. L. Whalen, *Biochim. Biophys. Acta - Proteins Proteomics* **2015**, *1854*, 1019–1037.

[3] P. Shannon, A. Markiel, O. Ozier, N. S. Baliga, J. T. Wang, D. Ramage, N. Amin, B. Schwikowski, I. Trey, *Genome Res.* **2003**, *13*, 2498–2504.

[4] F. Delaglio, S. Grzesiek, G. W. Vuister, G. Zhu, J. Pfeifer, A. Bax, *J. Biomol. NMR* **1995**, *6*, 277–293.

[5] J. Jumper, R. Evans, A. Pritzel, T. Green, M. Figurnov, O. Ronneberger, K. Tunyasuvunakool, R. Bates, A. Žídek, A. Potapenko, A. Bridgland, C. Meyer, S. A. A. Kohl, A. J. Ballard, A. Cowie, B. Romera-Paredes, S. Nikolov, R. Jain, J. Adler, T. Back, S. Petersen, D. Reiman, E. Clancy, M. Zielinski, M. Steinegger, M. Pacholska, T. Berghammer, S. Bodenstein, D. Silver, O. Vinyals, A. W. Senior, K. Kavukcuoglu, P. Kohli, D. Hassabis, *Nature* **2021**, *596*, 583–589.

[6] M. Mirdita, K. Schütze, Y. Moriwaki, L. Heo, S. Ovchinnikov, M. Steinegger, *Nat. Methods* **2022**, *19*, 679–682.

[7] J. Konc, D. Janežič, *Nucleic Acids Res.* **2014**, *42*, 215–220.

[8] F. H. Wallrapp, J. J. Pan, G. Ramamoorthy, D. E. Almonacid, B. S. Hillerich, R. Seidel, Y. Patskovsky, P. C. Babbitt, S. C. Almo, M. P. Jacobson, C. D. Poulter, *Proc. Natl. Acad. Sci. U. S. A.* **2013**, *110*, E1196–E1202.

[9] L. Holm, *Nucleic Acids Res.* **2022**, *50*, W210–W215.

[10] N. M. O'Boyle, M. Banck, C. A. James, C. Morley, T. Vandermeersch, G. R. Hutchison, *J. Cheminform.* **2011**, *3*, 1–14.

[11] J. Jung, T. Mori, C. Kobayashi, Y. Matsunaga, T. Yoda, M. Feig, Y. Sugita, *Wiley Interdiscip. Rev. Comput. Mol. Sci.* **2015**, *5*, 310–323.

[12] W. Humphrey, A. Dalke, K. Schulten, *J. Mol. Graph.* **1996**, *14*, 33–38.

[13] X. Robert, P. Gouet, *Nucleic Acids Res.* **2014**, *42*, 320–324.
